# Patient experience and barriers of using a mHealth exercise app in musculoskeletal (MSK) Physiotherapy

**DOI:** 10.1371/journal.pdig.0000626

**Published:** 2024-10-07

**Authors:** Jack Grodon, Christopher Tack, Laura Eccott, Mindy C. Cairns

**Affiliations:** 1 School of Health and Social Work, University of Hertfordshire, Hatfield, United Kingdom; 2 Musculoskeletal Physiotherapy Outpatients, Guy’s and St Thomas’ NHS Foundation Trust, London, United Kingdom; 3 Channel 3 Consulting, London, United Kingdom; Iran University of Medical Sciences, ISLAMIC REPUBLIC OF IRAN

## Abstract

Digital transformation has led to an abundance of digital health technologies (DHTs) readily available for Physiotherapists. In July 2020, the Physiotherapy department at a London NHS Trust implemented a mobile health (mHealth) exercise application (app), Physitrack. This service evaluation aims to evaluate patient experience and identify any barriers to using Physitrack/PhysiApp in musculoskeletal (MSK) Physiotherapy. An online experience survey was sent to 10,287 MSK Physiotherapy patients who had appointments between January 17th and April 9th 2022.The survey received 1,447 responses (response rate: 14.07%), with 954 (65.93%) respondents previously provided PhysiApp as part of their Physiotherapy management. Most participants used PhysiApp (83.06%), found it easy to use (82.20%) and had positive perceptions on how it added value to their Physiotherapy treatment through its functionality. However, negative impacts on patient-centred care and practical exercise demonstration were apparent in the qualitative results. Key barriers to use included suboptimal explanation, digital exclusion, registration/ login issues and opinion that PhysiApp was superfluous to Physiotherapy treatment. These differed to the main barriers of why participants stopped using/ used PhysiApp less: if they were confident exercising without it, their condition improved/ resolved, loss of motivation, their exercise programme ended or if they found their exercise programme was unsuitable. Despite multiple interdependent factors influencing patient experience and barriers of using PhysiApp, the survey results revealed the significant influence that is exerted by MSK Physiotherapists. The patient-physiotherapist interaction can positively or negatively impact upon many barriers of use and the subsequent potential added value of PhysiApp to MSK Physiotherapy treatment. Future research should focus on those at most risk of digital exclusion and health inequalities, exploring their barriers to using mHealth apps and other DHTs.

## Author summary

Physiotherapists commonly recommend home-based exercises for musculoskeletal (MSK) conditions, yet patient adherence remains a challenge, impacting treatment effectiveness and costs. With the rise of digital health technologies (DHTs), Physiotherapists now have access to tools like Physitrack, a mobile health (mHealth) exercise application (app). An online patient survey was sent to MSK Physiotherapy patients at Guy’s and St Thomas’ NHS Foundation Trust (GSTFT) in May 2022 to evaluate patient experience of using Physitrack and identify any barriers to use. Of the 954 respondents who reported they had previously been provided Physitrack, most participants found it easy to use and had positive perceptions on how it added value to their Physiotherapy treatment through its functionality. However, some participants revealed concerns regarding its impact on patient-centred care and exercise demonstration. Barriers to use included suboptimal explanation, digital exclusion, registration/ login issues and perceptions of Physitrack as unnecessary. Our evaluation reveals that the patient-physiotherapist interaction can have a significant influence on barriers to use and therefore the potential added value of Physitrack to Physiotherapy treatment. Future research should focus on people who are at most risk of digital exclusion and health inequalities, exploring the challenges they face in using mHealth apps and other DHTs.

## Introduction

Musculoskeletal (MSK) conditions represent the leading cause of disability worldwide (WHO, 2021). Physiotherapists commonly recommend home-based exercises to aid self-management of MSK conditions; however, adherence to these exercises is known to be suboptimal, which has implications for treatment effectiveness and costs [1–3].

Digital transformation of the National Healthcare Service (NHS) in the United Kingdom (UK) has been identified by the Department of Health and Social Care (DHSC) as a top priority [4]. There is currently an abundance of digital health technologies (DHTs) readily available for Physiotherapists to deliver home exercise programmes and encourage patient adherence [5,6]. In July 2020, the Physiotherapy department at Guy’s and St Thomas’ NHS Foundation Trust (GSTFT) implemented a mobile health (mHealth) exercise application (app), Physitrack. Physitrack operates two interconnected platforms: Physitrack, the clinician portal, and PhysiApp, the patient portal. Core features of the Physitrack platform used by clinicians include exercise prescription, patient education, patient reported outcome measures and telehealth. PhysiApp serves as the patient platform, which provides access to prescribed exercise programmes and advice through a website or app. In the first year of implementation, Physitrack was used by Physiotherapists to send out 17,000 exercise programs to patients across 20 different Physiotherapy teams, including MSK outpatients.

In response to this change in patient care at GSTFT, a service evaluation of patient experience and barriers to using a mHealth exercise app in MSK Physiotherapy was proposed using the Donabedian model for measuring healthcare quality [7]. Evaluation of patient experience is important for gauging the value of initiatives and optimising resource allocation, particularly in the face of NHS financial pressures [8]. Given the ongoing digital transformation within the NHS in the UK, the widespread adoption of mHealth apps is anticipated. Physitrack’s features (e.g. exercise videos and in-app reminders) can be mapped to the behaviour change technique taxonomy (BCTT) [9], indicating that using Physitrack has the potential to improve exercise adherence and treatment outcomes [6]. Whilst mHealth exercise apps such as Physitrack have the potential to be valuable adjuncts to Physiotherapy care, identifying and addressing any barriers limiting their use may improve their potential effectiveness.

Aim and Objectives

The aim was to understand patient experience of using a mHealth app in MSK Physiotherapy and identify any barriers to use. The objectives were to:

- Conduct a patient experience survey for patients who have had access to PhysiApp in MSK Physiotherapy at GSTFT
- Evaluate patient experience
- Identify any barriers to using PhysiApp

## Materials and methods

### Study design, area and period

A service evaluation encompassing a cross sectional study design was employed at GSTFT in London, England (UK), involving patients within the MSK Physiotherapy department. The study was completed from 14^th^ September 2021 to 25^th^ July 2022.

### Inclusion and exclusion criteria

Patients aged 16 or older, who had an MSK appointment (including initial assessment, follow-ups or group classes) via any method (face-to-face or remote) between 17^th^ January 2022 and 9^th^ April 2022 were eligible for inclusion. All sites offering MSK Physiotherapy under GSTFT were also encompassed in the study. Exclusion criteria included patients without a registered UK mobile number or those who had opted out of Trust surveys.

### Sampling and recruitment

Consecutive sampling was employed to achieve a heterogeneous sample representative of the population seen within the MSK Physiotherapy department at GSTFT. Recruitment was conducted from a centralised list of MSK patients with subgroup stratification not possible (e.g. patient’s first language). A Disclosure and Barring Service (DBS) check was completed to remove deceased patients (n = 16). The final sample contained 10, 311 patients.

### Survey design and provision

A survey was developed as there was no appropriate validated tool to collect the required data. The Donabedian model was used to assist in design and development [7]. The survey included ten closed questions with heterogeneity of question and response type. An open question (question 10) and text-box options for ’other’ answers allowed for qualitative insights and unexpected responses. Survey content was reviewed by subject matter experts (SMEs); a NIHR Research Fellow and the GSTFT Allied Health Professions (AHP) information officer, to aid content validity.

Piloting of the survey (n = 20) led to revisions in four questions and five item responses. The finalised survey was uploaded to a digital platform, *Civica*. Civica allowed all questions to be set as mandatory and also retained data from the surveys that were partially completed by participants. On the first page of the survey, a hyperlink directed participants to the Participant Information Sheet (PIS), which provided details about the study and was used to obtain consent.

The online survey was distributed via text message on 5^th^ May 2022 using a patient engagement platform (*Dr Doctor*) and remained open for 25 days, with a reminder message sent after two weeks.

### Data analysis

Survey results were exported into Microsoft Excel and securely stored on GSTFT servers for data protection. Quantitative data analysis utilised Excel to analyse all collected data, including partially completed surveys. Descriptive statistics, such as frequencies and counts, were employed as applicable, with no regression analysis performed. Secondary analysis of qualitative open text box entries labelled as ‘other’ was performed alongside another author.

Reflexive thematic analysis (TA) was completed on the open responses to question 10 using an inductive approach based on the most recent articulation of the six steps proposed by Braun and Clark [10], with a post-positive stance taken [11]. In alignment with reflexive TA, no investigator triangulation was performed on this question. Qualitative data was edited for major spelling and grammatical errors only.

### Ethics

Ethics approval for the service evaluation was granted by the University of Hertfordshire (HSK/PGT/UK/04963).

## Results

10,287 text messages were sent out successfully as 24 mobile numbers were invalid, with 1,447 survey responses received (14.07% response rate). 954 participants (65.93%) answered that they had previously been provided PhysiApp in an MSK appointment.

### Quantitative results

#### Participant demographics

Out of the 954 participants who reported they had previously been provided PhysiApp in an MSK appointment, 591 provided demographic information (61.95%), as shown in Table 1.

**Table 1:** Participant demographics for those provided PhysiApp in an MSK Appointment (n = 591).

Demographics	Survey responses
**Gender**	
Female	385 (65.14%)
Male	187 (31.64%)
Non-binary	3 (0.51%)
Transgender	1 (0.17%)
Prefer not to say	15 (2.54%)
**Age Group**	
16–24	8 (1.35%)
25–34	48 (8.12%)
35–44	87 (14.72%)
45–54	126 (21.32%)
55–64	162 (27.41%)
65–74	121 (20.47%)
75–84	34 (5.75%)
85+	5 (0.85%)
**Ethnicity**	
White British	228 (40.28%)
White Irish	16 (2.83%)
Any other White background	73 (12.9%)
Black Caribbean	43 (7.6%)
Black African	74 (13.07%)
Any other Black background	7 (1.24%)
Bangladeshi	6 (1.06%)
Chinese	6 (1.06%)
Indian	16 (2.83%)
Pakistani	5 (0.88%)
Any other Asian background	10 (1.77%)
White and Black Caribbean	5 (0.88%)
White and Black African	6 (1.06%)
White and Asian	4 (0.71%)
Any other mixed background	21 (3.71%)
Prefer not to say	36 (6.36%)
Other	10 (1.77%)
**Disabilities**	
Mobility difficulty	143 (23.99%)
Blind or partially sighted	2 (0.34%)
Deaf or hearing impaired	23 (3.86%)
Communication	6 (1.01%)
Learning disability	7 (1.17%)
Mental health condition	43 (7.21%)
I do not have a disability	300 (50.34%)
I would prefer not to say	43 (7.21%)
Other	29 (4.87%)

### Appointment type and method

Most respondents (67.71%) were provided PhysiApp access in their first Physiotherapy appointment, while 32.29% received access in a follow-up appointment. 71.21% of participants were provided PhysiApp access in a face-to-face appointment, 25.32% in a remote telephone appointment and 3.47% in a remote video appointment.

### Provision of home exercises by Physiotherapists

Most participants (36.45%) reported that their exercises were demonstrated to them face-to face, whilst 27.47% responded that they were told their exercises would be on PhysiApp. Participants also responded that they received exercise information through other methods such as having the exercises verbally described to them (15.86%), watching a video (e.g., YouTube), or being shown a picture of the exercises (10.11%). Only a small number of participants (1.25%) reported that they were not given any information.

### Information/Explanation for PhysiApp provided by Physiotherapists

Most respondents felt they were given the ‘right amount’ of information by Physiotherapists about what PhysiApp is (69.74%), its potential benefits (60.52%), and how to use it (63.47%). However, when it came to accessing help or support for any issues, only 45.57% of participants believed they received the ‘right amount’ of information and 31.55% stated no information was provided, as seen in Table 2.

**Table 2:** Information/ explanation for PhysiApp provided by Physiotherapists (n = 542).

Explanations	No information provided	Some information was provided but not enough	Right amount	Too much	I did not need information	Did not answer
**What** **PhysiApp is?**	58 (10.70%)	82 (15.13%)	378 (69.74% )	3 (0.55%)	21 (3.87%)	0 (0.00%)
**The possible benefits of using PhysiApp**	115 (21.22%)	57 (10.52%)	328 (60.52% )	8 (1.48%)	34 (6.27%)	0 (0.00%)
**How to use PhysiApp?**	80 (14.76%)	73 (13.47%)	344 (63.47% )	5 (0.92%)	40 (7.38%)	0 (0.00%)
**Where to get help or support if you had problems accessing or** **logging in to** **PhysiApp**	171 (31.55%)	61 (11.25%)	247 (45.57% )	2 (0.37%)	61 (11.25%)	0 (0.00%)

### Exercises/Information provided on PhysiApp by Physiotherapists

Most participants (81.65%) reported receiving the ‘right amount’ of exercises, while 8.75% found there were ‘too few’ and 9.61% considered receiving ‘too many.’ Regarding exercise difficulty, 69.74% deemed them ‘neither easy nor difficult,’ 15.13% ‘easy’, and 10.70% ‘very easy,’ with a minority finding them ‘difficult’ (0.55%) or ‘very difficult’ (3.87%). Pertaining to information about their condition, 53.33% felt they received the ‘right amount’, 20.18% reported receiving no information, and 18.95% considered the amount of information insufficient. Only 0.70% reported they received ‘too much’ information with 6.84% responding that they did not require any information about their condition.

### Participant experience with logging in and using PhysiApp

Most respondents (68.31%) reported being able to log in and use PhysiApp easily with

14.75% being able to log in but with some reported difficulty. Some patients did not log in to PhysiApp; either because they could not log in when they tried (4.37%) or did not attempt to log in (12.57%).

### Barriers to PhysiApp use

Table 3 shows the answers provided by participants for why they did not try to log in to PhysiApp, why participants were unable to log in to PhysiApp when they tried, or the login difficulties experienced by participants that went on to use PhysiApp.

**Table 3:** Barriers to PhysiApp use.

Answers	Participants that did not try to log in (number of responses, % of total) Total responses = 101	Participants that were unable to log in when they tried (number of responses, % of total) Total responses = 37	Participants that used PhysiApp but reported some difficulty when logging in (number of responses, % of total) Total responses = 144
**I was not told what PhysiApp is**	24 (23.76%)	4 (10.81%)	5 (3.47%)
**Other**	17 (16.83%)	4 (10.81%)	23 (15.97%)
**I did not have the digital skills to use PhysiApp**	12 (11.88%)	4 (10.81%)	18 (12.50%)
**I did not feel I needed to use PhysiApp to help me with my condition (e.g., the exercises or advice had already been explained/ demonstrated** **to me by the Physiotherapist)**	10 (9.90%)	1 (2.70%)	4 (2.78%)
**I did not receive an email with instructions of how to log in to PhysiApp**	9 (8.91%)	1 (2.70%)	9 (6.25%)
**I do not have access to** **internet/ Wi-Fi to use PhysiApp**	5 (4.95%)	1 (2.70%)	4 (2.78%)
**I was not told about the possible benefits of using PhysiApp**	5 (4.95%)	0 (0.00%)	6 (4.17%)
**I did not have access to a device to use PhysiApp on**	4 (3.96%)	2 (5.41%)	5 (3.47%)
**I did not have the motivation to use PhysiApp**	3 (2.97%)	2 (5.41%)	5 (3.47%)
**I did not have the time or opportunity to use PhysiApp**	3 (2.97%)	0 (0.00%)	2 (1.39%)
**I was not told where to get help or support to allow me to access/ log in to PhysiApp**	3 (2.97%)	1 (2.70%)	8 (5.56%)
**I was unable to download PhysiApp onto a device**	3 (2.97%)	3 (8.11%)	6 (4.17%)
**I was not given enough information on how to use PhysiApp**	2 (1.98%)	1 (2.70%)	30 (20.83%)
**I had concerns about PhysiApp storing and processing my personal data**	1 (0.99%)	3 (8.11%)	1 (0.69%)
**I tried to log in, but the access code/ year of birth did not allow me to access PhysiApp**	0 (0.00%)	10 (27.03%)	11 (7.64%)
**I did not use PhysiApp as it was not available in my preferred language**	0 (0.00%)	0 (0.00%)	1 (0.69%)

The responses with the highest scores differed among the three groups: ‘I was not told what PhysiApp is’ (23.76%), ‘I tried to log in, but the access code/year of birth did not allow me to access PhysiApp’ (27.03%), and ‘I was not given enough information on how to use PhysiApp’ (20.83%) were the most commonly reported reasons, respectively.

### Participant perceptions of PhysiApp

For the participants that used PhysiApp, Table 4 demonstrates that most users agreed or strongly agreed that PhysiApp helped achieve their personal treatment goals (55.12%), complete their exercises regularly as per the Physiotherapist’s recommendations (61.89%) and perform their exercises with the correct technique (77.18%). Furthermore, 82.20% of respondents agreed or strongly agreed that PhysiApp was easy to use.

**Table 4:** Participant perceptions of PhysiApp (n = 517).

Perceptions	Strongly Agree	Agree	Neither agree nor disagree	Disagree	Strongly Disagree	Don’t know
**PhysiApp helped me to achieve my personal goals**	109 (21.08%)	176 (34.04 %)	130 (25.14%)	46 (8.90%)	37 (7.16%)	19 (3.67%)
**PhysiApp helped me to do my exercises regularly as per the** **Physiotherapist’s** **Recommendations**	123 (23.79%)	197 (38.10 %)	108 (20.89%)	48 (9.29%)	27 (5.22%)	14 (2.71%)
**PhysiApp reminders helped prompt me to complete my exercises**	114 (22.05%)	165 (31.91 %)	110 (21.28%)	56 (10.83%)	35 (6.77%)	37 (7.16%)
**PhysiApp was a good way to keep a diary/ record of the exercises I completed**	134 (25.92%)	179 (34.62 %)	107 (20.70%)	50 (9.67%)	21 (4.06%)	26 (5.03%)
**PhysiApp helped me to do my exercises with the correct technique**	157 (30.37%)	242 (46.81 %)	72 (13.92%)	29 (5.61%)	13 (2.51%)	4 (0.77%)
**PhysiApp was easy to use**	176 (34.04%)	249 (48.16 %)	57 (11.03%)	26 (5.03%)	5 (0.97%)	4 (0.77%)

### Barriers to continued use of PhysiApp

A total of 40.20% of participants reported continuing to use PhysiApp according to their Physiotherapist’s recommendations, while 26.20% were using it less frequently than recommended. PhysiApp was no longer in use by 33.60% of participants. Reasons for reduced or discontinued use of PhysiApp are outlined in Table 5.

**Table 5:** Barriers to continued use of PhysiApp.

Answers	Using PhysiApp less (number of responses, % of total) Total responses = 164	Stopping using PhysiApp (number of responses, % of total) Total responses = 231
**I was confident I could complete the exercises without continuing to use PhysiApp**	52 (31.71%)	47 (20.35%)
**Other**	31 (18.90%)	39 (16.88%)
**I did not have the motivation or** **discipline to continue using** **PhysiApp**	16 (9.76%)	15 (6.49%)
**I got better and no longer needed to use PhysiApp**	14 (8.54%)	26 (11.26%)
**I did not have the time or opportunity to continue using** **PhysiApp**	9 (5.49%)	8 (3.46%)
**I started a different type of treatment for my condition**	8 (4.88%)	15 (6.59%)
**I trialled the exercises on PhysiApp but did not feel they were suitable for my condition**	8 (4.88%)	20 (8.66%)
**I had technical issues with PhysiApp**	6 (3.66%)	8 (3.46%)
**I stopped using PhysiApp when my exercise programme ended**	5 (3.05%)	22 (9.52%)
**I did not have the confidence in my** **ability to exercise at home on my own**	4 (2.44%)	9 (3.90%)
**I forgot I had PhysiApp downloaded on my device**	3 (1.83%)	7 (3.03%)
**I used a different method of viewing exercises to help my condition**	3 (1.83%)	4 (1.73%)
**I found PhysiApp too difficult to use**	2 (1.22%)	3 (1.30%)
**I had technical issues with the device I used to access PhysiApp**	2 (1.22%)	2 (0.87%)
**I had too many notifications/ reminders for PhysiApp on my device**	1 (0.61%)	3 (1.30%)
**I deleted PhysiApp to free up memory on my device**	0 (0.00%)	2 (0.87%)
**I had concerns about PhysiApp storing and processing my personal data**	0 (0.00%)	2 (0.87%)

### Device types used to access PhysiApp

Most users (72.95%) reported accessing PhysiApp via a mobile phone, 13.40% of participants used computers or laptops, 10.42% accessed PhysiApp through tablet devices and 3.23% reported using other types of devices.

### Duration of PhysiApp use

Duration of PhysiApp use prior to discontinuation was varied. A summary of the findings is as follows: 18.18% of participants used PhysiApp for less than a week, while 10.91% used it for 1 to 2 weeks. 20.61% continued using the app for more than 2 weeks but less than 4 weeks, 13.94% for 4 to 6 weeks, and 36.36% for more than 6 weeks.

### Qualitative results

**Fig 1:**
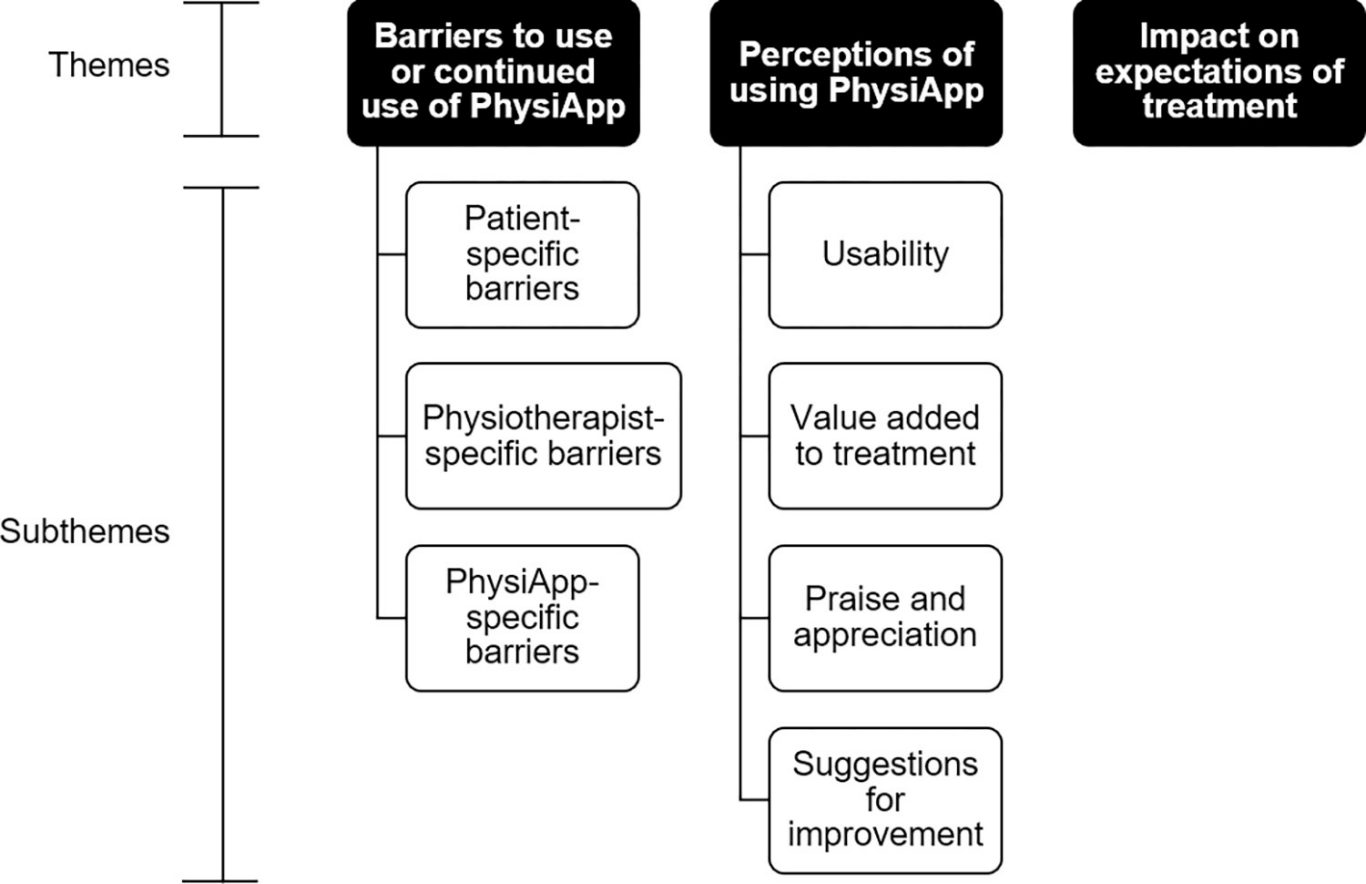
Qualitative themes and subthemes.

Two hundred and seventy-six responses were provided to ‘If you have any other comments about your experience of using PhysiApp please share them below’. From this data three themes evolved. These were ‘***barriers to use or continued use of PhysiApp’***; ‘***perceptions of using PhysiApp’*** and ‘***impact on expectations of treatment’***. Qualitative themes and subthemes are outlined in Fig 1.

### Theme 1: ‘Barriers to use or continued use of PhysiApp’

Participants provided comments on barriers to use or continued use of PhysiApp. From this data three subthemes were identified, the first being ‘patient-specific barriers’. This included a lack of digital skills, accessibility to digital devices, data privacy concerns, preferences for other methods of exercise prescription and opinions that PhysiApp was superfluous to treatment:

*“A very good app once you know how to access and use it. Firstly I wasn’t sure but my children showed me how to use it and download it”*

*(Participant 668)*

*“I would have preferred a link to online resources which do not request or hold my personal information”*

*(Participant 417)*

There was also patient–specific barriers to continued use such as stopping use when their condition improved or when they were confident of how to complete their exercises, condition severity and other ‘life barriers’:

*“I found it hard to use as frequently when I began to feel stronger”*

*(Participant 1131)*

*“I was going through a tough time at the same time that I was using the app so I didn’t use it as often as I should”*

*(Participant 1416)*

The second sub-theme ‘Physiotherapist-specific barriers’ included non-provision of PhysiApp, suboptimal PhysiApp explanation/demonstration, inappropriate exercises, unclear explanation of exercise dosage and suboptimal set-up of exercise programme length:

*“The exercises were too light and too simple and did not help with my condition. I don’t believe they were appropriate or suitable to treat my condition”*

*(Participant 883)*

*“Apps like these become successful if enough information about its purpose and rationale are provided prior…”*

*(Participant 1123)*

The final sub-theme ‘PhysiApp-specific barriers’ included technical issues, issues when logging in and exercises programmes on PhysiApp not updating:

*“I can only reiterate that I tried to access the PhysiApp program by following the instruction*

… nothing happened, even after repeated efforts”

*(Participant 412)*

*“…There were technical issues with changing the exercises after my appointment” (Participant 715)*

### Theme 2: ‘Perceptions of using PhysiApp’

Participants provided their perceptions of using PhysiApp as part of their Physiotherapy care and the added value it had on their treatment. The four subthemes were:

**‘Usability’**

Participants responded that PhysiApp was easy to use:

*“Well thought out, easy to use…”*

*(Participant 563)*

*“…PhysiApp is an easy tool to use…”*

*(Participant 1007)*

**‘Value added to Physiotherapy treatment’**

Participants reported how the functionality of the PhysiApp (exercise videos, pain monitoring, diary and reminders) added value to their Physiotherapy treatment with regards to reducing hospital visits, helping achieve treatment goals, increasing motivation and supporting exercise adherence:

*“…the videos are always there to reassure me I’m doing exercises properly…”*

*(Participant 460)*

*“…I do not live local to physio and would have had problems attending face-to-face sessions but this clearly shows you what to do. I think it is fabulous!”*

*(Participant 662)*

**‘Praise and appreciation’**

The data showed that participants praised and appreciated access to PhysiApp:

*“It is a great app. I enjoyed using it and it was very beneficial to me”*

*(Participant 1164)*

*“Appreciate it & great idea”*

*(Participant 1274)*

**‘Suggestions for improvement’**

Suggestions to improve PhysiApp included the comments section, follow-along exercise videos, advanced reporting, language availability and the chat function:

*“You guys should include an online personal trainer element to the videos, kind of like the YouTube tutorials where the instructor does all the reps and encourages you as you do them too…”*

*(Participant 1029)*

### Theme 3: ‘Impact on expectations of treatment’

Participants reported that PhysiApp appeared to have diminished their experience of the core tenets of MSK Physiotherapy they expected with regards to face-to-face demonstration of exercises and patient-centred care:

*“It’s become my physiotherapist. Using the app takes you away from a professional which isn’t great. The explanations are fine so are the videos but you cannot be sure you’re doing them correctly or if they are right for you…”*

*(Participant 587)*

## Discussion

The results provide an understanding of the patient experience of using an mHealth app as an adjunct to MSK Physiotherapy and identified barriers to using this technology. Both quantitative and qualitative data indicate positive perceptions of experience; including ease of use, enhancement of treatment, increased motivation, supported exercise adherence, facilitation of treatment goal achievement, and reduced hospital utilisation. These usability findings align with previous studies [6,12]. However, qualitative data also indicated that such technology can be in contradiction to patient expectations for patient-centred care and practical demonstration of exercises. Further barriers to use were identified which are to be discussed further. The Donabedian “structure-process-outcome” model provides the framework for further interpretation.

The Donabedian model is a framework for evaluating healthcare quality, consisting of three main components: structure, process, and outcomes [7]. “Structure" is concerned with the resources and infrastructure necessary to deliver services, which can impact care quality. In this instance, the type and method of appointment impact the experience of participants. Most participants (67.71%) received PhysiApp access during an initial appointment, likely to meet their expectations for personalised advice [13,14]. However, it is likely that the time requirement to set up such technologies [15–17] may explain why 32.29% of respondents obtained PhysiApp access during follow-up appointments [18,19]. The provision of PhysiApp in face-to-face appointments (71.21%) rather than remote appointments, may indicate that greater opportunity to *onboard* individuals in-person [20], minimising barriers to use.

The Donabedian domain of “process” refers to the interactions between healthcare professionals (HCPs) and patients, reflecting the delivery of care quality. Within this service evaluation this concerns the manner Physiotherapists deployed the app alongside traditional exercise demonstration, how they framed the utility of the app with Physiotherapy treatment, and what content was shared within the app to describe prescribed exercise programmes. Physiotherapists demonstrated exercises for 36.45% of participants, whilst 27.47% were left reliant upon PhysiApp. Remote appointments may inhibit physical exercise demonstration; however, it is possible that PhysiApp may have become a default facilitator of exercise provision for some Physiotherapists to organisational factors such as time, equipment accessibility and space [21,22] or individual factors such as knowledge and competency [21,23]. An explanatory qualitative study by Danbjorg et al. [24] to develop a DHT for osteoarthritis found that participants valued contact with HCPs for exercise technique correction and reassurance, which they postulated was key in improving patient competence and self-efficacy. It is argued that absence of physical exercise demonstration has the potential to impact self-efficacy and subsequently be a barrier to engagement to both exercise and PhysiApp. Qualitative feedback corroborated concerns about diminished patient-centred care and lack of face-to-face exercise demonstration of exercises.

The findings from both the quantitative (Table 2) and the qualitative data (the ‘Physiotherapist specific barriers’ subtheme) revealed a lack of optimal description and explanation of the utility of PhysiApp to participants prior to their use. An ethnographic qualitative study by Keel et al. [17] previously identified ‘micro-’ and ‘meso-level factors’ contributing to suboptimal PhysiApp explanation. ‘Micro-level’ factors included sufficient digital literacy of Physiotherapists to ensure competence to use the app, as well as to motivate and support reluctant patients. At ‘meso-level’, training was vital to allow Physiotherapists to understand the app’s functionality, subsequently allowing this understanding to be transferred to patients. Like Keel et al. [17] there is no protected Physitrack/ PhysiApp training time in the MSK department alongside unclear digital competence of Physiotherapists, which may have impacted these results.

The provision of content through PhysiApp can relate to the amount, type and difficulty of exercises prescribed. Most respondents (81.65%) reported receiving the “right amount” of exercises. Provision of too many exercises is associated with low adherence [25], while too few may have the opposite effect [26]. Thus, exercise volume was unlikely to be a barrier to use for most participants. Similarly, most patients (69.74%) found the overall difficulty of exercises ‘neither easy nor difficult,’ while 25.83% considered them ‘easy’ or ‘very easy’. Exercise difficulty can be influenced by factors such as non-demonstration, clinician competence, or reluctance to encourage patients to exercise into pain [21,27]. Of the responses provided by participants regarding their reasons for using PhysiApp less than recommended or discontinuing its use, the unsuitability of exercises accounted for only 4.27% and 7.79% of the total answers, respectively. The results suggest that exercise difficulty was not a significant barrier to the use of PhysiApp. Nonetheless, it remains an important factor for Physiotherapists to consider.

Careful consideration of exercise prescription variables by Physiotherapists is crucial for optimal utilisation and continued use of PhysiApp. Physiotherapist-specific factors such as competence and experience, as well as the practical demonstration of exercises, which can be affected by organisational and individual factors, will likely have influence over this.

The “outcome” domain of the Donabedian model relates to the effects of healthcare services on patients, and here focuses on whether PhysiApp was used by patients. A total of 124 respondents (16.94%) did not login to Physitrack; either because they could not log in when they tried (4.37%) or did not attempt to login (12.57%). Failure to log-in, was related to technical issues, such as not receiving email instructions (8.91%), and issues related to inputting access codes (27.03%). PhysiApp’s setup process requires manual entry of the patient’s details, leaving risks of human error. Registration and login difficulties with DHTs have similarly been identified as barriers in previous systematic reviews [28,29].

Alongside technical issues, the second most common reason cited for inability to access the technology was that respondents did not have the digital skills to use the app (Table 3). This relates to the multi-dimensional phenomenon of digital exclusion [30]. Up to 9% of residents, in the two main boroughs within which this MSK Physiotherapy department provides care, are digitally excluded [31,32]. Digital skills of patients were similarly raised by Keel et al. [17] as being a barrier at ‘micro-level’ which was intrinsically linked to lack of access to a digital device. These barriers have also been identified by other authors [16,17,28,29,33] and could be mediated by the explanation and demonstration of PhysiApp to participants by the Physiotherapist and confirmation of digital device and internet access.

Similar to the systematic review by Svendsen et al. [29], the survey findings did not indicate that data privacy concerns were a major barrier to use. Conversely, Keel et al. [17] did highlight that such concerns regarding information governance were a barrier to app use. It is possible that since the completion of this service evaluation, the uptake of technology usage following the COVID-19 pandemic may have influenced participant responses in this area.

The primary barrier for participants not attempting to log in was the lack of understanding regarding the app’s benefit and utility (23.76%), while 9.90% of answers provided cited not perceiving a need for PhysiApp to address their condition. These findings underscore the significance of patient-physiotherapist interactions in educating and preparing patients to effectively use the app and highlight the importance of emphasising the app’s relevance in addressing their specific condition.

Conversely, other results relate to respondents who did use PhysiApp. The device most used to access PhysiApp was a mobile phone (72.95%). Qualitative data revealed that the accessibility and convenience of PhysiApp on mobile phones was advantageous to participants, however also demonstrated that some participants’ mobile devices could be incompatible or inconvenient, posing a barrier to PhysiApp use. Physiotherapists should ensure patients are aware of alternative device options for accessing PhysiApp to minimise this as a barrier. Most participants who used the app found the process to log in easy (82.24%) and found PhysiApp easy to use (82.2%). Two other studies [6,12] have used the System Usability Scale (SUS) [34] when evaluating PhysiApp, resulting in high scores of 85.5 and 79.2 (out of 100). According to the interpretation by Bangor et al. [34] this indicates the system is ‘highly usable’ and ‘good to excellent’ respectively. Usability can present a significant barrier to use DHTs [16,28,29]; however, in line with these two studies the survey results did not identify this as a barrier. Future mHealth apps should place emphasis on user-friendly design and gather feedback from users for iterative improvements to ensure usability is not a barrier to effective engagement and usage.

Similarly, respondents who used PhysiApp found specific functionalities of the technology beneficial. For example, diary keeping with the app was found useful by most participants, in line with other studies [5,15,35,36]. DHTs featuring interactive graphs of exercise adherence have been shown to enhance belief in competence and motivation [37,38]. Reminders were found helpful by 53.96% of users, contrasting with the findings from a cross-sectional study on a mHealth app for pelvic floor muscles [39], where 75% of respondents rated this feature 8 or higher out of 10 (10 = really like). The discrepancy in survey findings may have been influenced by the suboptimal explanation/ demonstration of PhysiApp to participants, which was also identified in the qualitative data. The exercise videos within the app, were felt by 77.18% of respondents to assist the performance of exercise with correct technique; with qualitative data supporting this finding and highlighting the usefulness of exercise videos. This is consistent with other studies showing positive participant feedback using DHTs with exercise videos or visual aids [3,40,41] and the study by Bennell et al. [6], where a mean score of 4.0 out of 5 (5 = strongly agree) was given to *‘PhysiApp was helpful in helping me carry out my exercises*’ by respondents.

These three features (exercise diary, reminders, and videos) can be mapped to the BCTT [8] to support patient adherence. It could be argued that these functions contributed to participants either ‘strongly agreeing’ or ‘agreeing’ that PhysiApp helped them complete their exercises regularly as per the Physiotherapist’s recommendations (61.89%) and that PhysiApp helped them achieve their personal treatment goals (55.12%). PhysiApp’s added value to Physiotherapy treatment is demonstrated by both quantitative results (Table 4) and qualitative data. However, inhibitors to realising this value depend on the explanation/ demonstration of PhysiApp provided by Physiotherapists, as well as the multifactorial factors related to exercise adherence [1]. Previous studies have demonstrated that mHealth apps can improve exercise adherence compared to standard Physiotherapy care; however, limitations of these studies include limited follow-up, recall bias [6], and the use of supplementary phone calls or motivational text messages [5].

The third most common reason for reduced PhysiApp use was a lack of motivation or discipline to continue using the app (9.76%). Explanations for this are likely multifaceted and overlap with factors related to adherence to home-based exercise [1], as acknowledged by other systematic reviews [28,29]. A lack of interest in technology and motivation to improve health through DHT usage are known barriers to DHT engagement, however it has been demonstrated that this can be mitigated by enhancing patient understanding of DHT benefits and HCP support for use [16,17,28,29], further emphasising the importance of the patient physiotherapist interaction.

Despite exercise being the most common intervention provided by Physiotherapists [42–44], unsupervised exercise or exercise as a treatment modality may not meet patient expectations or treatment preferences [42,45]. Of the reasons provided for participants reducing or discontinuing their use of PhysiApp, starting a different type of treatment for their condition accounted for 4.88% and 6.59% of the total answers, respectively.

### Limitations & methodological considerations

The survey achieved a 61.95% completion rate based on the completion of the last survey question on demographics. This is comparable to a recent online survey of similar length (10 minutes) which had a completion rate of 59% [46] and may also have allowed for non-response bias. It was not possible to account for when participants were first provided with PhysiApp which could have introduced recall bias to the results.

The low response rate (14.07%) allowed for non-response bias and compares unfavourably to the average 46% online survey response rate seen in a recent systematic review of survey studies [47]. The study aimed for a large and diverse sample to ensure representativeness to the local population, however, if Physiotherapists assumed patients’ digital literacy and affinity for digital tools, this may have led to some patients unjustly missing being provided PhysiApp and their views being underrepresented [17]. It is also acknowledged that the survey topic and lack of access to a smartphone (with internet) may have contributed to non-response bias and further underrepresented views of those with less affinity for technology [48].

The internal validity of the survey could have been enhanced by quantifying expert judgment from subject matter experts (SMEs) with indices such as the Content Validity Index (CVI) [49] and integrating validated tools like the SUS [34]. The SUS, which has been incorporated into other studies evaluating PhysiApp, would have allowed for greater comparison of results [6,12]. However, it was not selected due to its length (10 questions) and reduced reliability when used on a sample that is not comprised of first-time system users [50]. The survey also included questions with multiple answers, which could have introduced recency or primacy bias secondary to ordering [51].

The background of the main author as being the primary catalyst for the implementation of Physitrack into the Physiotherapy department at GSTFT should also be acknowledged.

### Conclusions

This service evaluation successfully explored patient experience of using PhysiApp in MSK Physiotherapy and identified barriers to use. Results revealed that most participants used PhysiApp, found it easy to use, and had positive perceptions of how it enhanced their Physiotherapy treatment through its functionality aligning with previous studies [6,12]. However, qualitative feedback did indicate show that PhysiApp can negatively impact on patient experience of the core aspects of MSK Physiotherapy treatment that are typically expected, such as patient-centred care and practical demonstration of exercises.

Key barriers for why participants did not use PhysiApp were identified as; suboptimal instruction on the use of PhysiApp, digital exclusion, technical issues and patient views that PhysiApp was superfluous to Physiotherapy treatment. The main barriers for why participants discontinued PhysiApp use were; confidence exercising without it, their condition improved/ resolved, they lost motivation, their exercise programme ended or they found their exercise programme was unsuitable.

Multiple interdependent factors can influence patient experience and barriers to using PhysiApp. MSK Physiotherapists play a vital role in patient experience of using PhysiApp and their interaction with patients can positively or negatively impact upon barriers of use and the potential added value of PhysiApp to MSK Physiotherapy treatment. To support successful integration of PhysiApp as an adjunct of Physiotherapy treatment it is recommended that clinicians have protected training time on their use, allowing them to become fully competent with the app, and subsequently this can be explained more comprehensibly to patients. The role of PhysiApp as an adjunct to care and it’s benefits should be discussed with patients alongside practical demonstration of exercises to support individualised care. Finally, clinicians should be educated to explore digital exclusion with patients to establish suitability of app provision, and offer signposting to local digital access support schemes if needed.

As DHTs become more commonplace [20], future research should evaluate patient experience and barriers to the use of technology. Research should focus on those who are at most risk of digital exclusion and health inequalities, exploring their barriers to using mHealth apps and other DHTs.
